# Can immunization with BCG delay the onset of Alzheimer's disease?

**DOI:** 10.1111/ggi.14680

**Published:** 2023-09-21

**Authors:** Ofer N. Gofrit, Charles L. Greenblatt, Benjamin Y. Klein, Tamir Ben‐Hur, Herve Bercovier

**Affiliations:** ^1^ Department of Urology Hadassah – Hebrew University Medical Center Jerusalem Israel; ^2^ Department of Microbiology and Molecular Genetics Hebrew University‐Hadassah Faculty of Medicine Jerusalem Israel; ^3^ Department of Neurology Hadassah–Hebrew University Medical Center Jerusalem Israel

Dear Editor,

Alzheimer's disease (AD) affects about 10% of the population older than 65 years in developed countries and its prevalence is expected to rise as population ages. Thus far, there are no available therapies to treat or prevent AD efficiently. Given the doubling in burden prevalence every 5 years, postponement of AD onset by just 5 years would have a dramatic effect of the 50% reduction in burden of disease on society. Neuroinflammation is a major player in the pathogenesis of AD and a potential target for intervention. Epidemiological and preclinical studies suggest that the tuberculosis vaccine, bacillus Calmette‐Guérin (BCG), which is also an immunomodulator can potentially do that. A highly significant correlation between BCG immunization and the prevalence of AD was found in 166 countries with available information.^1^ BCG is also given intravesicaly to patients with bladder cancer to reduce the recurrence of non‐muscle invasive disease. Despite the intravesical route, this treatment has well‐documented systemic effects that can theoretically modulate the immune system. The peak incidence of bladder cancer is at the 6–8 decades of life, in parallel to the accumulation of AD pathology at its preclinical stage. Thus, patients with bladder cancer can serve as a natural laboratory for examining the effect of BCG on the risk of developing AD.

Thus far, five retrospective studies examined the effect of intravesical BCG on the odds ratio of dementia in patients with bladder cancer from different ethnologic backgrounds, both in community settings and in tertiary centers (Table 1).^2^, ^3^, ^4^, ^5^, ^6^ These studies included 19 983 BCG treated and 27 222 untreated patients. All studies showed significant decrease in the odds ratio of dementia in patients treated with BCG. The study with the longest follow‐up showed the most significant decrease (by 79%).^3^ Two studies also documented a dose‐dependent relation between the number of the BCG instillations and the effect on dementia.^2^, ^5^

**Table 1:** AD incidence among populations of BCG‐treated or‐untreated patients with bladder cancer

Data source	Tertiary center^2^	Tertiary center^3^	Tertiary center^4^	HMO^5^	HMO^5^	SEER database^6^
No. of patients, BCG; *n* (%)	3388 (52.4)	878 (64)	319 (24.7)	1587 (23.5)	315 (6.6)	13 496 (50.8)
No. of patients, no BCG; *n* (%)	3078 (47.6)	493 (36)	971 (75.3)	5147 (76.5)	4445 (93.4)	13 088 (49.2)
Age^†^ (years), BCG; mean (SD)	69.9 (9.3)	67.5 (12.6)	69.0 (11.9)	72.8 (7.5)	69.1 (12.3)	76.7 (6.6)
Age^†^ (years), no BCG; mean (SD)	70.7 (10)	69.0 (13.4)	71.5 (12.0)	73.9 (8.1)	70.4 (12.2)	79.3 (7.7)
Follow‐up time (years), BCG; mean (SD)	6.8 (4.3)^‡^	8 (3, 14)^§^	3 (2, 6)^§^	7.2 (3.7)^‡^	3.3 (1.4)^‡^	Not specified
Follow‐up time (years), no BCG; mean (SD)	7.0 (4.6)^‡^	8 (3, 14)^§^	3 (1, 6)^§^	7.2 (4.2)^‡^	4.0 (2.4)^‡^	Not specified
AD, BCG; *n* (%)	202 (5.9)	21 (2.4)	10 (3.1)	75 (4.8)	0 (0)	964 (7.1)
AD, no BCG; *n* (%)	262 (8.5)	44 (8.9)	89 (9.2)	336 (6.5)	75 (4.8)	1228 (9.4)
HR (95% CI)	0.8 (0.66–0.96)	0.21 (0.12–0.35)	0.41 (0.21–0.80)	0.73 (0.57–0.94)	Not specified	0.73 (0.67–0.79)^¶^
*P*‐value	0.02	<0.0001	<0.0001	0.015	0.008	<0.05
Remark	Overall survival benefit		Multiethnic population	Dose dependency		Dose dependency

AD, Alzheimer's disease; CI, confidence interval; BCG, bacillus Calmette‐Guérin; HR, hazard ratio.

^†^ Age at diagnosis of bladder cancer.

^‡^ Average (SD).

^§^ Median (IQR).

^¶^ Adjusted for age, sex, T‐stage, and Charlson Comorbidity Index.

How can BCG given intravesicaly decrease the occurrence of AD? BCG induces different local and systemic responses. The local response to BCG is massive. After the fourth instillation there is an increase in urine levels of interleukin (IL)‐1β, IL‐2, and IL‐6 (>1000‐fold), tumor necrosis factor‐alpha, interferon‐gamma, and macrophage colony‐stimulating factor (100‐fold). In the serum, there is a 10‐fold increase in IL‐2 levels and a three‐fold increase in BCG‐induced killer cell activity of peripheral blood mononuclear cells.^7^ IL‐2 at low levels expands the population of CD4+ CD25+ Foxp3+ regulatory T cells, which decrease neuroinflammation and delay the onset of AD.^8^, ^9^ BCG, which is usually given to patients with bladder cancer who are mentally sound, will take effect in the preclinical stage of AD and can potentially slow its clinical onset.

So, can BCG delay the onset of AD? Despite the consistent results the answer is: we still do not know. All reports were retrospective and are therefore, subject to several potential biases included the following.

1. Selection bias; BCG could have been given only to more fit patients.
2. Bias due to the high percentage of censored data; more than 90% of the patients were censored in all study groups.
3. Bias due to the retrospective design of the study and reliance on coding systems.

Thus, only a randomized double‐blind trial can provide a definitive answer on the effect of BCG on AD. Considering the consistency of the retrospective data, it seems that time has come for such a trial.

## Disclosure statement

The authors declare no conflict of interest.

## Data Availability

Data sharing not applicable to this article as no datasets were generated or analysed during the current study.
